# Correction: Residue-based propensity of aggregation in the Tau amyloidogenic hexapeptides AcPHF6* and AcPHF6

**DOI:** 10.1039/d2ra90058k

**Published:** 2022-06-08

**Authors:** Abha Dangi, Abhishek Ankur Balmik, Archana Kisan Ghorpade, Nalini Vijay Gorantla, Shweta Kishor Sonawane, Subashchandrabose Chinnathambi, Udaya Kiran Marelli

**Affiliations:** Central NMR Facility, CSIR-National Chemical Laboratory Dr HomiBhabha Road 411008 Pune India; Division of Organic Chemistry, CSIR-National Chemical Laboratory Dr HomiBhabha Road 411008 Pune India; Academy of Scientific and Innovative Research (AcSIR) Ghaziabad-201002 India; Neurobiology Group, Division of Biochemical Sciences, CSIR-National Chemical Laboratory Dr HomiBhabha Road 411008 Pune India

## Abstract

Correction for ‘Residue-based propensity of aggregation in the Tau amyloidogenic hexapeptides AcPHF6* and AcPHF6’ by Abha Dangi *et al.*, *RSC Adv.*, 2020, **10**, 27331–27335, https://doi.org/10.1039/D0RA03809A.

The authors regret that the one of the affiliations (affiliation *c*) was incorrectly shown in the original manuscript. The corrected list of affiliations is as shown here.

The Royal Society of Chemistry apologises for these errors and any consequent inconvenience to authors and readers.

## Supplementary Material

